# Precision Pharmacotherapy for Childhood Obesity: Molecular Targets Beyond Single GLP-1 Receptor Agonism and a Framework for Disease Modification

**DOI:** 10.3390/ijms27209269

**Published:** 2026-10-19

**Authors:** Hajar Dauleh, Khalid Hussain

**Affiliations:** Division of Endocrinology, Department of Pediatric Medicine, Sidra Medicine, Doha PO BOX 26999, Qatar; hdauleh1@sidra.org

**Keywords:** pediatric obesity, precision medicine, pharmacotherapy, disease modification, GLP-1 receptor agonist, incretin polyagonism, amylin, FGF21, melanocortin, monogenic obesity

## Abstract

Childhood obesity is a heterogeneous chronic disease shaped by genetic susceptibility, neuroendocrine signaling, energy homeostasis, adipose dysfunction, insulin resistance, and environmental exposure. Glucagon-like peptide-1 (GLP-1) receptor agonists have markedly improved treatment efficacy, but heterogeneous response, weight recurrence after withdrawal, residual organ risk, and limited long-term pediatric data expose the limitations of a single-pathway, weight-centered model. This narrative review integrates the molecular rationale and clinical evidence for precision pharmacotherapy beyond single GLP-1 receptor agonism. It examines glucose-dependent insulinotropic polypeptide (GIP)/GLP-1 and GLP-1/glucagon co-agonism, triple agonism, amylin combinations, fibroblast growth factor 21 (FGF21) analogues, leptin replacement, and melanocortin-4 receptor (MC4R) agonism, separating established pediatric evidence from adult proof-of-concept and preclinical inference. Genotype-directed treatment for monogenic and syndromic obesity and lesion-directed treatment for acquired hypothalamic obesity provide the clearest examples of causal pathway correction. For common obesity, treatment selection should integrate developmental stage, hyperphagia, insulin resistance, ectopic fat, body composition, genetic context, treatment burden, and early response. Disease modification should be evaluated across growth, puberty, bone and lean tissue, beta cell and hepatic health, physical function, psychosocial well-being, safety, and durability. A dynamic cycle of phenotyping, mechanism-based treatment, multidomain monitoring, adaptation, and longitudinal re-phenotyping may improve the clinical relevance of pediatric obesity care.

## 1. Introduction

Childhood obesity is a chronic, relapsing disease with biologically defended body weight rather than a simple consequence of individual choice. Globally, obesity prevalence among children and adolescents aged 5–19 years increased approximately fourfold between 1990 and 2022, and severe obesity has become more common in many regions [1]. A high body mass index (BMI) in childhood often persists into adulthood, particularly when obesity is severe or continues through adolescence [2,3]. The clinical burden extends beyond body size. Children may develop insulin resistance, dyslipidemia, hypertension, obstructive sleep apnea, polycystic ovary syndrome, impaired mobility, psychosocial distress, and metabolic dysfunction-associated steatotic liver disease (MASLD); youth-onset type 2 diabetes can also progress rapidly [4,5,6].

Modern pharmacotherapy has changed treatment expectations. In the STEP TEENS trial, once-weekly semaglutide produced a mean 16.1% reduction in BMI at 68 weeks, compared with a 0.6% increase with placebo, and improved several cardiometabolic risk markers [7]. Liraglutide and extended-release phentermine/topiramate have also demonstrated efficacy in adolescents [8,9]. These results support earlier integration of pharmacotherapy within comprehensive pediatric obesity care rather than prolonged reliance on lifestyle intervention alone [10].

The efficacy of glucagon-like peptide-1 (GLP-1) receptor agonism also exposes unresolved questions. Some children have little or no clinically meaningful response, others discontinue treatment because of adverse effects or access barriers, and weight commonly recurs after withdrawal. BMI reduction does not identify which biological component has improved; visceral fat, hepatic fat, insulin sensitivity, beta cell stress, inflammation, lean mass, fitness, and quality of life may change differently. In addition, the dominant molecular driver may be impaired melanocortin signaling, congenital leptin deficiency, hypothalamic injury, adipose dysfunction, or severe insulin resistance rather than the incretin axis. Precision treatment therefore requires a broader therapeutic repertoire and more informative patient characterization.

This review develops a molecular-to-clinical framework for precision pharmacotherapy in childhood obesity. Beyond GLP-1 refers both to progression from single-receptor GLP-1 agonists toward combination and non-incretin targets and to evaluation beyond weight loss as the sole therapeutic objective. We distinguish established pediatric evidence from adult proof-of-concept and preclinical inference because efficacy and safety cannot be assumed across developmental stages. Section 3.1, Section 3.2, Section 3.3, Section 3.4 and Section 3.5 establish central appetite circuits, gut–brain–pancreas signaling, adipose dysfunction, insulin resistance, and developmental context. Section 4 reviews established medicines and GLP-1 response heterogeneity. Section 5.1, Section 5.2, Section 5.3, Section 5.4, Section 5.5, Section 5.6 and Section 5.7 examine GIP/GLP-1, GLP-1/glucagon and triple agonism, amylin combinations, FGF21 analogues, leptin replacement, MC4R agonism, and additional targets. Section 6, Section 7 and Section 8 connect genotype and hypothalamic lesions to phenotyping and multidomain outcomes. Section 9, Section 10 and Section 11 present the proposed clinical framework, safety and equity considerations, and priorities for mechanistic and clinical validation.

## 2. Literature Search and Scope

This is a narrative, state-of-the-art review rather than a systematic review. The authors searched PubMed through 12 August 2026 using combinations of childhood obesity, adolescent obesity, pharmacotherapy, precision medicine, GLP-1, GIP, glucagon, amylin, FGF21, leptin, melanocortin, setmelanotide, monogenic obesity, hypothalamic obesity, body composition, MASLD, insulin resistance, multi-omics, and treatment response. A targeted update of the peer-reviewed literature and current United States prescribing information was completed on 19 September 2026. Priority was given to randomized trials, prospective pediatric studies, regulatory studies, consensus guidance, and mechanistic human studies. Adult trials were included only when they establish target validity or define an emerging therapeutic class without adequate pediatric data. Conference abstracts and company announcements were not treated as definitive efficacy evidence. Regulatory indications vary by jurisdiction and may change. For this revision, targeted source checks on 9 October 2026 addressed reviewer-suggested publications and prescribing information; this was not a repeat systematic search. Figure 1, Figure 2 and Figure 3 are conceptual illustrations, not patient images or experimental data. Figure 1, Figure 2 and Figure 3 were generated using ChatGPT (6.1,OpenAI). The authors reviewed the figures for scientific accuracy and take full responsibility for their final content.

## 3. Childhood Obesity as a Heterogeneous Molecular Disease

The following subsections separate central control of intake from peripheral nutrient handling while recognizing their interaction. The same BMI can arise from different combinations of these mechanisms; developmental context determines which interventions and outcomes are clinically appropriate. Figure 1 provides a conceptual map of these entry points, without implying that mechanistic plausibility validates pediatric prescribing.

### 3.1. Defended Energy Balance and Central Appetite Circuits

Body weight is regulated by interacting homeostatic, hedonic, and cognitive systems. Within the arcuate nucleus, pro-opiomelanocortin (POMC) neurons generate alpha-melanocyte-stimulating hormone, which activates melanocortin-4 receptors (MC4R) in downstream neurons and promotes satiety and energy expenditure. Agouti-related peptide/neuropeptide-Y neurons oppose this signal and increase feeding. Leptin and insulin communicate long-term energy stores, while gut-derived peptides provide meal-related information. These circuits interact with brainstem vagal pathways, mesolimbic reward networks, stress responses, sleep, and executive control [11,12].

Weight loss activates compensatory biology: hunger increases, satiation weakens, and energy expenditure falls beyond that expected from the reduction in body mass. These endocrine adaptations can persist after weight reduction and favor regain [13]. Pharmacotherapy works partly by counteracting this mechanism. The dominant mechanism differs among children. Relentless, food-seeking hyperphagia from early life suggests disruption of leptin–melanocortin signaling; rapid weight gain after a suprasellar tumor or its treatment suggests that acquired hypothalamic obesity and reward-driven eating in an adverse environment may reflect a different balance of homeostatic and hedonic mechanisms. These phenotypes can overlap, but they are not interchangeable.

### 3.2. Gut–Brain–Pancreas Signaling

GLP-1 and glucose-dependent insulinotropic polypeptide (GIP) are nutrient-responsive incretin hormones. GLP-1 enhances glucose-dependent insulin secretion, suppresses glucagon under hyperglycemic conditions, slows gastric emptying, and acts in central and vagal circuits to reduce intake. GIP also stimulates insulin secretion, but its role in energy balance is context-dependent. Chronic GIP receptor agonism can complement GLP-1 agonism, whereas experimental GIP antagonism can also reduce weight, suggesting that receptor timing, cellular desensitization, and central versus adipose actions matter [14,15].

Amylin is co-secreted with insulin by pancreatic beta cells. It slows gastric emptying, suppresses postprandial glucagon, and signals satiation through the area postrema and related hindbrain circuits. Native amylin aggregates and has a short half-life; engineered analogues overcome these limitations. Glucagon, conversely, increases hepatic glucose production but also promotes satiety, lipolysis, amino acid turnover, and energy expenditure. Combining glucagon activity with sufficient incretin action may capture these benefits while limiting hyperglycemia. These complementary physiological actions provide the rationale for dual and triple agonists.

### 3.3. Adipose Tissue Expandability, Inflammation, and Ectopic Fat

BMI is an imperfect proxy for the biological consequences of adiposity. When subcutaneous adipose tissue cannot safely accommodate surplus energy through adipocyte hyperplasia and remodeling, hypertrophic adipocytes develop hypoxia, fibrosis, altered adipokine secretion, and immune cell recruitment. Lipids then accumulate in the liver, skeletal muscle, pancreas, and visceral depots. In adolescents, hepatic and intramyocellular fat and adipose inflammation are strongly linked to insulin resistance, independent of total adiposity [16,17].

The adipose expandability model helps explain why children with the same BMI may have markedly different cardiometabolic risk. A child with severe insulin resistance, hypertriglyceridemia, and MASLD may need therapy that improves ectopic lipid handling even if weight reduction is modest. A child with marked hyperphagia and relatively preserved metabolic health may instead need dominant appetite pathway control. Adiponectin, triglyceride-rich lipoproteins, liver fat, visceral-to-subcutaneous fat distribution, and inflammatory markers are candidate stratifiers, but few have been prospectively validated to guide the selection of an anti-obesity medication.

### 3.4. Insulin Resistance, Beta Cell Stress, and Metabolic Inflexibility

Puberty transiently reduces insulin sensitivity, which amplifies risk in genetically susceptible youth. Initially, beta cells compensate by increasing insulin secretion. Persistent insulin resistance, ectopic fat, glucolipotoxicity, inflammation, and genetic susceptibility can eventually produce inadequate compensation and dysglycemia. Youth-onset type 2 diabetes is characterized by rapid beta cell deterioration and early complications, making prevention a particularly important disease-modifying goal [6,18].

Metabolic flexibility, the ability to switch between lipid and carbohydrate oxidation in response to fasting, feeding, and activity, may also be impaired. Therapies that improve insulin sensitivity, reduce hepatic fat, or increase energy expenditure could therefore add value beyond appetite suppression. This is the biological rationale for investigating glucagon co-agonism, fibroblast growth factor 21 (FGF21) analogues, and combinations that target both energy intake and nutrient handling.

### 3.5. Developmental Context

Children are not small adults. Linear growth, pubertal development, bone accrual, neurodevelopment, fertility, family dependence, and school participation are active biological and social processes. BMI trajectories are age- and sex-dependent; BMI z-score compresses at the upper tail and may be less responsive in severe obesity. A percentage change in BMI or a percentage in the 95th BMI percentile can be more informative, but neither captures body composition. Appetite suppression that produces rapid weight reduction may also reduce protein and micronutrient intake. Pediatric trials must therefore measure height velocity, pubertal progression, bone health, menstrual function, nutritional adequacy, strength, and lean mass alongside adiposity. Figure 1 integrates the principal biological signals and therapeutic entry points relevant to this developmental context. Figure 1 distinguishes leptin–POMC–MC4R signaling from GLP-1, GIP, and amylin modulation of appetite and satiation. The gut–pancreatic and hepatic panels locate incretin, amylin, and FGF21 signals. Glucagon is discussed in Section 5.2 as a complementary hepatic target but is not explicitly depicted in the unchanged original figure. These pathways interact rather than form independent treatment categories. The drug label legend distinguishes indication-specific pediatric use from investigational strategies. Evidence supporting these therapeutic entry points includes incretin physiology [11,14,15], MC4R-directed treatment [19,20,21,22], FGF21 analogues [23,24,25], and leptin replacement [26,27,28,29]. Leptin replacement must be interpreted in its specialist, diagnosis-specific regulatory context.

**Figure 1:**
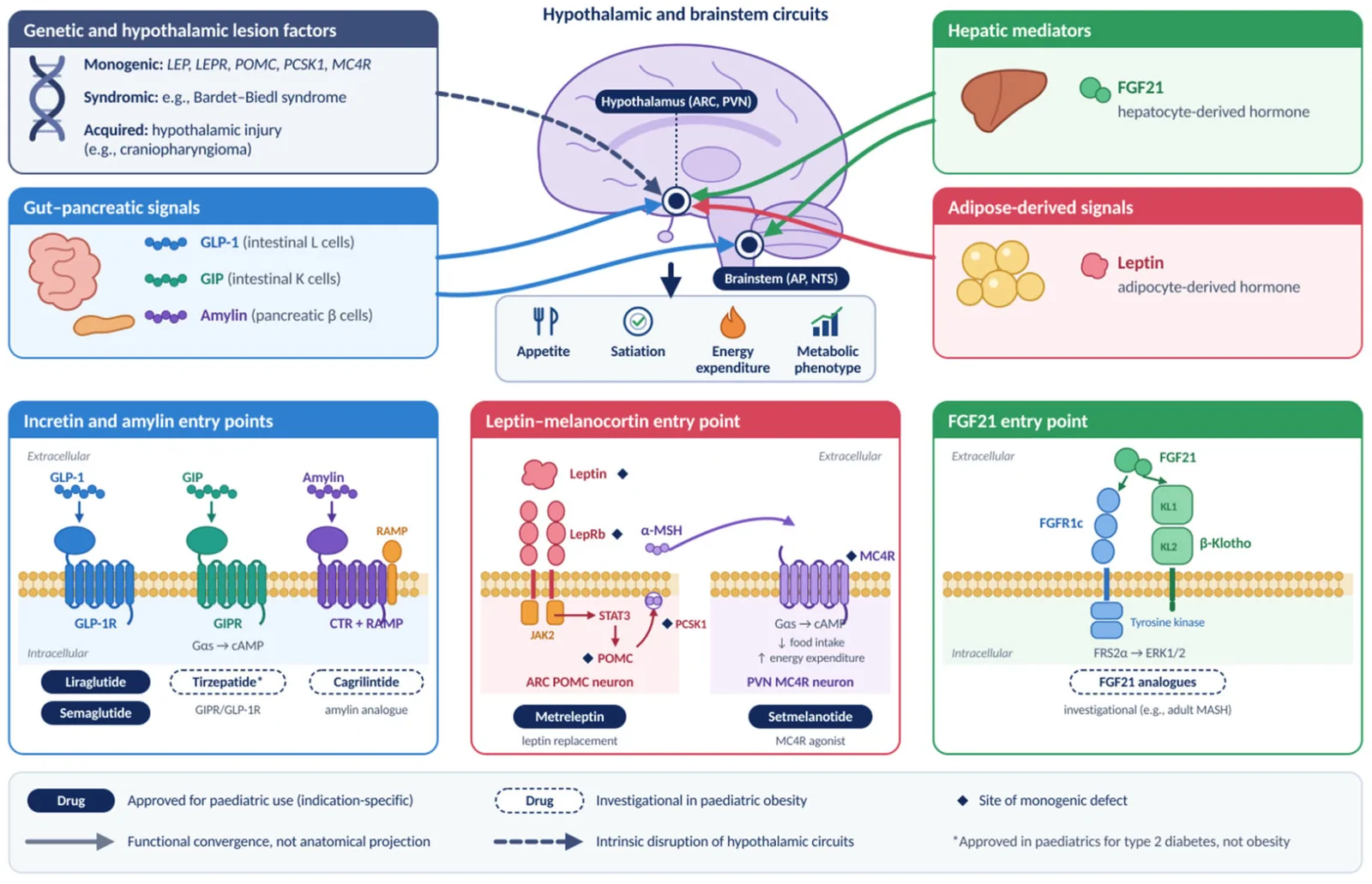
Mechanistic architecture and therapeutic entry points in childhood obesity. Genetic and acquired hypothalamic factors, gut–pancreatic peptides, FGF21, and leptin converge on hypothalamic and brainstem circuits regulating appetite, satiation, energy expenditure, and metabolic phenotype. Lower panels show therapeutic entry points. Solid and dashed drug labels indicate indication-specific pediatric approval and investigational use, respectively; dashed arrows denote intrinsic circuit disruption; diamonds mark monogenic defects. Abbreviations: α-MSH, alpha-melanocyte-stimulating hormone; AP, area postrema; ARC, arcuate nucleus; cAMP, cyclic adenosine monophosphate; CTR, calcitonin receptor; FGF21, fibroblast growth factor 21; FGFR1c, fibroblast growth factor receptor 1c; GIP, glucose-dependent insulinotropic polypeptide; GLP-1, glucagon-like peptide-1; JAK2, Janus kinase 2; MC4R, melanocortin-4 receptor; NTS, nucleus tractus solitarius; PCSK1, proprotein convertase subtilisin/kexin type 1; POMC, pro-opiomelanocortin; PVN, paraventricular nucleus; RAMP, receptor activity-modifying protein; STAT3, signal transducer and activator of transcription 3. Sources by pathway: incretin signaling [11,14,15]; MC4R-directed treatment [19,20,21,22]; FGF21 analogues [23,24,25]; leptin replacement [26,27,28,29].

## 4. Current Pharmacotherapy and the Limits of a GLP-1-Centered Paradigm

### 4.1. Established Options

Current United States options for nonsyndromic pediatric obesity include orlistat for adolescents aged 12 years and older, phentermine for short-term use in patients older than 16 years, extended-release phentermine/topiramate from age 12, liraglutide from age 12 in those meeting the labeled weight criterion, and injectable semaglutide from age 12. A randomized trial also demonstrated liraglutide efficacy in children aged 6 to <12 years, but trial evidence should not be conflated with a regulatory indication [30]. Setmelanotide is mechanistically distinct and is discussed separately. Regulatory indications differ by region, and treatment should be embedded in multicomponent care [10,31,32,33,34]. Table 1 summarizes the established and selected targeted options.

**Table 1:** Established and selected targeted pharmacotherapies relevant to pediatric obesity.

Therapy	Principal Mechanism	Evidence and Current Role	Main Precision Considerations	Dose and Titration
Orlistat	Gastrointestinal lipase inhibition [35]	Modest average effect; limited by gastrointestinal adverse effects and fat-soluble vitamin concerns [35]	May fit a family preferring non-central therapy; weak choice for severe hyperphagia or high treatment-intensity needs [10,35]	Age ≥ 12 years: 120 mg orally with each fat-containing main meal, up to three times daily; omit if the meal is missed or contains no fat [35].
Phentermine	Sympathomimetic appetite suppression [36]	Short-term option for older adolescents in the United States [36]	Avoid or use cautiously with uncontrolled hypertension, tachyarrhythmia, marked anxiety, stimulant misuse risk, or pregnancy [36]	Age > 16 years, short-term use: dose individualized to the lowest effective dose; the cited tablet/capsule label gives 37.5 mg orally daily, with 18.75 mg daily sufficient for some patients. Formulation and renal function affect dosing [36].
Phentermine/topiramate ER	Catecholaminergic appetite suppression plus GABA/glutamate and appetite effects [9,34]	Substantial BMI reduction in a 56-week adolescent trial [9,34]	Teratogenic risk demands pregnancy prevention; monitor mood, cognition, heart rate, and blood pressure [34]	Age ≥ 12 years: 3.75/23 mg orally daily for 14 days, then 7.5/46 mg daily. After 12 weeks, <3% BMI reduction prompts 11.25/69 mg for 14 days, then 15/92 mg daily; <5% BMI reduction after 12 weeks on that dose requires discontinuation with gradual taper [34].
Liraglutide 3.0 mg	Daily GLP-1 receptor agonism [8,33]	Superior to placebo in adolescents; mean effect smaller than semaglutide and response is heterogeneous [8,37]	Daily injection, gastrointestinal tolerance, gallbladder risk, and early response trajectory influence continuation [33,37]	Age ≥ 12 years and weight >60 kg: 0.6 mg subcutaneously daily, increasing weekly by 0.6 mg to 3.0 mg daily; pediatric escalation may take up to 8 weeks. Maintenance is 3.0 mg, or 2.4 mg if 3.0 mg is not tolerated; discontinue if 2.4 mg is not tolerated. Stop if BMI falls <1% after 12 weeks at maintenance [33].
Semaglutide 2.4 mg	Weekly GLP-1 receptor agonism [7,32]	Largest placebo-controlled mean BMI reduction among established nonsyndromic pediatric medicines [7]	Useful when strong appetite suppression and glycemic benefits are desired; preserve nutrition and lean mass; plan for long-term access [32]	Age ≥ 12 years: 0.25, 0.5, 1.0, and 1.7 mg subcutaneously weekly, each for 4 weeks; then 2.4 mg weekly (recommended) or 1.7 mg weekly according to response and tolerance. Escalation may be delayed by 4 weeks when not tolerated [32].
Setmelanotide	MC4R agonism [19,38]	Pathway-directed therapy for defined genetic obesity and acquired hypothalamic obesity [19,20,21,22,38]	Requires correct molecular or clinical diagnosis; monitor pigmentation, mood, sexual adverse effects, and indication-specific endocrine risks [38]	Subcutaneous daily dosing differs by diagnosis. BBS or POMC/PCSK1/LEPR deficiency: age ≥ 12—start 2 mg for 2 weeks then 3 mg; age 6–<12—start 1 mg for 2 weeks, then 2 mg and up to 3 mg if tolerated. Acquired hypothalamic obesity, age ≥ 6: 0.5, 1, and 2 mg, each for 2 weeks, then 3 mg. Younger eligible children start on 0.5 mg; maintenance is weight-based. Consult label tables for younger children and renal impairment [38].

Dose note: Table 1 summarizes standard regimens for the cited United States indications; it is not a complete prescribing guide. Renal impairment, interruption/re-initiation, and age/weight-specific setmelanotide regimens require the full current label. All medicines are adjuncts to comprehensive care. ER, extended release; GABA, gamma-aminobutyric acid; MC4R, melanocortin-4 receptor.

### 4.2. Heterogeneity of Response

Mean trial results conceal wide response distributions. In STEP TEENS, semaglutide shifted the full response curve and enabled many adolescents to move below the obesity threshold, yet not every participant achieved clinically important BMI reduction [39]. A post hoc analysis of SCALE Teens likewise demonstrated heterogeneous liraglutide response, with early weight change providing more practical information than most baseline characteristics [37]. Real-world response is further shaped by adherence, dose escalation, adverse effects, food environment, sleep, concurrent weight-promoting medication, mental health, family resources, and continuity of coverage.

A recent pediatric network meta-analysis reinforces the value of combining pharmacotherapy with lifestyle treatment for short-term adiposity outcomes, while emphasizing the need for longer comparative studies [40]. Indirect comparisons across different populations, trial durations, and supportive interventions do not establish an individual prescribing hierarchy or long-term developmental safety.

Inadequate response should prompt a structured review rather than attribution to willpower. Clinicians should confirm therapeutic exposure, determine whether adverse effects are compromising nutrition, reassess hyperphagia and the possibility of genetic or hypothalamic obesity, and identify concurrent medications or circumstances that may counteract treatment. Precision medicine also cannot be reduced to a one-time genetic test; serial treatment response is itself an informative biomarker.

The adult literature also supports studying individualized incretin titration. Vangoitsenhoven et al. discuss personalized dose escalation, but their commentary is not pediatric trial evidence [41]. In FLEX-SEMA, a retrospective adult cohort showed clinically relevant improvements with variable maintenance doses; dose selection was influenced by response, adverse effects, and cost [42]. Its nonrandomized design cannot establish dose equivalence, superior safety, or pediatric cost-effectiveness. Pediatric practice should follow current product-specific titration and stopping rules, delaying escalation when permitted by the label and clinically appropriate; lower doses should not be assumed equally effective. Trials should test whether response-guided titration preserves benefit while reducing treatment burden, nutritional risk, and cost [32,33].

### 4.3. Chronicity and Weight Recurrence

Adult withdrawal trials show substantial weight recurrence after semaglutide and tirzepatide discontinuation, accompanied by the loss of some cardiometabolic improvements [43,44,45]. In the adolescent liraglutide trial, BMI z-score increased more after treatment was stopped than after placebo [8]. These observations support obesity pharmacotherapy as long-term treatment for many patients rather than a short course that permanently resets physiology.

Chronic treatment raises pediatric questions that remain incompletely answered: cumulative exposure across puberty, pregnancy planning, bone and lean-mass effects, long-term gallbladder and pancreatic safety, mental health, adherence fatigue, transition to adult care, and the consequences of involuntary interruption. Disease modification should therefore not be defined as “cure after withdrawal”. A more realistic definition is durable alteration of high-risk biology and life-course trajectory, which may require continued therapy.

## 5. Emerging Molecular Targets Beyond Single GLP-1 Receptor Agonism

These strategies expand the molecular target repertoire, but their translational status differs. The following subsections separate adult target validation from pediatric clinical evidence and identify developmental outcomes that must be assessed before routine use.

### 5.1. Dual GIP/GLP-1 Receptor Agonism

Tirzepatide integrates GIP and GLP-1 receptor agonism in one molecule. In adults without diabetes, the SURMOUNT-1 trial demonstrated mean weight reductions of up to approximately 21% at 72 weeks, and a subsequent head-to-head trial showed greater weight reduction with tirzepatide than semaglutide [46,47]. Mechanistically, GLP-1 contributes to appetite suppression and glucose-dependent insulin secretion, while GIP may enhance insulin action, modulate adipose nutrient handling, and engage complementary central circuits [14].

This class warrants pediatric evaluation, particularly in adolescents with severe obesity and insulin resistance. However, as of August 2026, the United States obesity label states that safety and effectiveness have not been established in pediatric patients [48]. Adult efficacy cannot establish effects on growth, puberty, eating behavior, lean mass, or long-term beta cell health in adolescents. Tirzepatide should therefore be presented as a high-priority pediatric research strategy rather than an established pediatric obesity therapy.

### 5.2. GLP-1/Glucagon and GIP/GLP-1/Glucagon Agonism

Adding glucagon receptor agonism aims to increase energy expenditure and hepatic lipid mobilization, while GLP-1 and/or GIP activity restrains appetite and hyperglycemia. Survodutide, a GLP-1/glucagon co-agonist, produced substantial weight loss in an adult phase 2 trial [49]. Retatrutide activates GIP, GLP-1, and glucagon receptors; in an adult phase 2 obesity trial, the highest doses produced mean weight reductions exceeding 20% at 48 weeks [50]. A liver fat substudy showed marked reductions in hepatic fat, supporting a metabolic effect that may extend beyond reduced energy intake [51].

For pediatrics, these agents are attractive for phenotypes combining severe obesity, MASLD, hypertriglyceridemia, and insulin resistance. They also introduce specific uncertainties. Glucagon activity can raise glucose, heart rate, and amino acid turnover; rapid weight loss may threaten lean tissue; and the optimal receptor balance for a growing child may differ from that for an adult. Trials should incorporate MRI-derived liver fat, stable-isotope or clamp measures in mechanistic subsets, body composition, protein adequacy, and physical function rather than relying only on BMI.

### 5.3. Amylin Analogues and Incretin–Amylin Combinations

Amylin provides meal-related satiation through pathways that are partly distinct from GLP-1. Cagrilintide is a long-acting amylin analogue with once-weekly dosing. It produced dose-dependent weight loss in adults, and combination with semaglutide was designed to amplify satiation while using complementary receptors [52,53]. In the phase 3 REDEFINE program, co-administered cagrilintide–semaglutide produced clinically important weight reductions in adults with and without type 2 diabetes [54,55].

An amylin-based strategy may be particularly relevant when incomplete satiation, large meal sizes, or a plateau on GLP-1 therapy dominates. Whether baseline eating-behavior phenotypes predict response remains unproven. Pediatric development should evaluate nausea, vomiting, nutritional intake, hypoglycemia in diabetes, and lean mass, as well as whether stronger satiation improves or worsens disordered eating. The field also needs trials that compare rational switching with combination therapy rather than simply adding agents indefinitely.

### 5.4. FGF21 Analogues: Targeting Metabolic Quality Rather than Appetite Alone

Fibroblast growth factor 21 (FGF21) is an endocrine regulator of nutrient stress, lipid oxidation, insulin sensitivity, and adiponectin signaling. Pharmacological analogues have shown effects on liver fat, triglycerides, and fibrosis-related outcomes in adults with metabolic steatohepatitis. Efruxifermin improved fibrosis and steatohepatitis endpoints in the phase 2b HARMONY study, and pegozafermin improved fibrosis in ENLIVEN [23,24]. These agents generally produce less weight loss than modern incretin polyagonists, but their ability to improve hepatic and lipid biology may be the more important attribute [25].

This distinction informs precision care. A child with MASLD and severe dyslipidemia may derive disease-modifying benefit from liver- and adipose-directed therapy even if BMI changes modestly, whereas FGF21 monotherapy would be unlikely to control severe hyperphagia. No established pediatric obesity indication exists, and skeletal growth, bone turnover, pubertal physiology, and long-term safety require dedicated study. Combination with appetite-targeting therapy is biologically plausible but must be tested for additive benefit.

### 5.5. Leptin Replacement

Leptin concentrations are high in most common obesity, reflecting leptin resistance, and additional leptin is therefore not a general anti-obesity treatment. Congenital leptin deficiency is the crucial exception. Affected children present with severe early-onset hyperphagia, rapid weight gain, endocrine and immune abnormalities, and low or inappropriately normal leptin. Recombinant leptin replacement can produce dramatic and sustained reductions in hunger and fat mass and can restore neuroendocrine function [26,27,28].

Congenital leptin deficiency provides a direct example of precision pharmacotherapy: a biochemical and molecular diagnosis identifies hormone deficiency, replacement addresses the causal defect, and benefit extends beyond body weight. The example also shows why rare and common obesity cannot be collapsed into one mechanism. Metreleptin has an established role in generalized lipodystrophy, where leptin deficiency drives severe insulin resistance and hypertriglyceridemia, but the therapeutic and regulatory context differs from congenital leptin-deficient obesity [29].

### 5.6. Melanocortin-4 Receptor Agonism

Setmelanotide activates MC4R downstream of several leptin–melanocortin defects. Phase 3 studies established reductions in hunger and weight in patients with biallelic POMC/PCSK1 or LEPR deficiency and in Bardet–Biedl syndrome [19,20]. The VENTURE study extended evidence to children aged 2–5 years, in whom early correction of pathological hyperphagia may reduce cumulative exposure to obesity [21].

The therapeutic reach of MC4R agonism expanded in 2026. A randomized phase 3 trial in acquired hypothalamic obesity demonstrated substantially greater BMI and hunger reductions with setmelanotide than placebo, and current United States labeling includes patients aged 4 years and older with acquired hypothalamic obesity [22,38]. This is conceptually important: precision therapy can be directed by an acquired circuit lesion, not only by germline genotype.

Response should not be extrapolated to every MC4R pathway gene or to common polygenic obesity. Heterozygous MC4R deficiency is the most common monogenic obesity, with variable penetrance and functional severity [56]. Some variants retain partial signaling, and therapeutic response may depend on residual receptor function; setmelanotide cannot correct absent or non-functional MC4R itself. Functional variant interpretation and indication-specific evidence are therefore essential.

### 5.7. Additional Targets on the Horizon

Other strategies include peptide YY analogues, ghrelin pathway modulation, GDF15–GFRAL agonism, cannabinoid pathway modulation with peripherally restricted compounds, myostatin/activin inhibition to preserve lean mass, and agents that alter mitochondrial energy efficiency. Each addresses a plausible component of obesity biology, but development has often been limited by nausea, cardiovascular or neuropsychiatric toxicity, compensatory pathways, or insufficient efficacy. Mechanistic plausibility alone is insufficient in children; a new target must combine a favorable developmental safety profile with clinically meaningful benefits beyond established therapies. Table 2 summarizes the evidence boundary for the leading emerging strategies.

**Table 2:** Emerging molecular strategies beyond single GLP-1 receptor agonism.

Molecular Strategy	Dominant Biological Actions	Most Plausible Phenotype	Evidence Boundary and Priority Outcomes
GIP/GLP-1 agonism	Appetite reduction, insulin secretion, insulin sensitivity, adipose nutrient handling [14,46,47]	Severe obesity with insulin resistance or dysglycemia [14,46]	Strong adult evidence; pediatric efficacy and developmental safety needed [46,47,48]
GLP-1/glucagon or triple agonism	Appetite reduction plus energy expenditure and hepatic lipid mobilization [49,50,51]	Severe obesity with MASLD, visceral adiposity, or hypertriglyceridemia [51]	Adult phase 2/3 development; monitor glucose, heart rate, amino acid metabolism, and lean mass [49,50,51]
Amylin analogues ± GLP-1	Satiation, smaller meal size, slower gastric emptying, glucagon suppression [52,53,54,55]	Incomplete satiation or plateau despite incretin therapy [52,53]	Adult phase 3 evidence; pediatric nutrition, tolerability and eating-behavior studies needed [54,55]
FGF21 analogues	Hepatic fat reduction, lipid oxidation, adiponectin signaling, insulin sensitivity [23,24,25]	MASLD or atherogenic dyslipidemia disproportionate to BMI [23,24,25]	Adult liver trials; pediatric hepatic, bone, and growth outcomes needed [23,24,25]
Leptin replacement	Corrects leptin deficiency, reduces hyperphagia, restores endocrine signaling [26,27,28,29]	Congenital leptin deficiency; selected lipodystrophy [26,29]	Highly targeted established biology; confirm biochemical and molecular diagnosis [26,27,28,29]
MC4R agonism	Restores downstream melanocortin signaling, reduces hunger, may increase energy expenditure [19,20,21,22,38]	POMC/PCSK1 or LEPR deficiency, Bardet–Biedl syndrome, acquired hypothalamic obesity [19,20,21,22,38]	Indication-specific clinical evidence; functional diagnosis and safety monitoring required [38]

Abbreviations: GIP, glucose-dependent insulinotropic polypeptide; GLP-1, glucagon-like peptide-1; FGF21, fibroblast growth factor 21; MC4R, melanocortin-4 receptor; POMC, pro-opiomelanocortin; PCSK1, proprotein convertase subtilisin/kexin type 1; LEPR, leptin receptor; MASLD, metabolic dysfunction-associated steatotic liver disease. Evidence note: references identify the mechanistic, trial, or guidance basis for each entry. Phenotype matching, advanced monitoring, and disease-modifying interpretations are proposed syntheses, not source-validated pediatric prescribing rules. Adult-only evidence is not a pediatric recommendation.

## 6. Genotype-Directed and Lesion-Directed Treatment

### 6.1. When to Suspect a Rare Biological Driver

Genetic investigation should be considered when severe obesity begins before the age of 5 years, particularly when accompanied by intense hyperphagia, rapid crossing of weight percentiles, a family history of severe early-onset obesity, or consanguinity. Additional clues include developmental delay, retinal dystrophy, polydactyly, hearing loss, hypogonadism, adrenal insufficiency, recurrent diarrhea, immune abnormalities, renal disease, or dysmorphic features [57,58]. A normal developmental examination does not exclude monogenic obesity. These features align with endocrine guidance recommending genetic evaluation in selected children with extreme early-onset obesity and hyperphagia [59].

### 6.2. Therapeutically Relevant Disorders

Testing may include a curated obesity gene panel, chromosomal microarray when syndromic features are present, and exome or genome sequencing when first-line testing is unrevealing. Interpretation must connect genotype to phenotype and inheritance. A variant of uncertain significance (VUS) does not establish causality in isolation. Interpretation should consider segregation, population frequency, phenotype, inheritance, and functional evidence. The setmelanotide label permits selected POMC, PCSK1, or LEPR variants classified as VUS when interpreted in the clinical context; this indication-specific provision should not be generalized to other genes or therapies [38,59]. Table 3 summarizes the principal clinical clues, mechanism-informed treatments, and limitations for therapeutically relevant genetic and acquired disorders.

**Table 3:** Genotype- and lesion-directed treatment opportunities.

Disorder or Pathway	Characteristic Clues	Targeted or Mechanism-Informed Treatment	Key Limitations
Congenital leptin deficiency (*LEP*)	Severe infantile hyperphagia, low/inappropriately normal leptin, infections, or hypogonadism [26,27,28,29]	Recombinant leptin replacement [26,27,28,29]	Access and regulatory pathway vary. Product-specific immunogenicity and hematological warnings from the lipodystrophy context should not be presented as demonstrated congenital LEP-deficiency risks [29].
*POMC* or *PCSK1* deficiency	Early hyperphagia; POMC may include adrenal insufficiency and pale/red hair; PCSK1 may include neonatal diarrhea and multiple endocrinopathies [19,57,59]	Setmelanotide for eligible molecular diagnoses; replace endocrine deficits [19,38]	Phenotypic and variant interpretation can be complex [57,59]
*LEPR* deficiency	Severe early hyperphagia, hypogonadotropic hypogonadism, immune/endocrine features [19,57,59]	Setmelanotide for eligible diagnoses [19,38]	Requires adequate downstream MC4R signaling [19,38]
Bardet–Biedl syndrome	Retinal dystrophy, polydactyly, renal disease, hypogonadism, developmental features [20,57]	Setmelanotide; organ-specific multidisciplinary care [20,38]	Response varies; visual and renal disease continue to require direct management [20,57]
Heterozygous *MC4R* deficiency	Early-onset obesity, hyperphagia, tall stature/greater lean mass in some patients [56,57]	Structured obesity pharmacotherapy; clinical trials and functional stratification [56,57]	MC4R agonism cannot overcome complete receptor loss; no universal genotype-specific treatment [38,56,57]
16p11.2/SH2B1, *SIM1*, *ADCY3,* and other rare disorders	Neurodevelopmental, behavioral, or endocrine clues vary [57,58,60]	Mechanism-informed standard therapy and trials [57,60]	Evidence is limited; avoid treating a VUS as causal [59,60]
Prader–Willi syndrome	Neonatal hypotonia followed by hyperphagia, endocrine deficits, characteristic behavior [57,59]	Growth hormone and endocrine replacement; rigorous food security; selected symptom-directed pharmacotherapy [57,59]	Multisystem disease requires specialist care; this row does not provide a complete 2026 hyperphagia-drug regulatory inventory [57,59].
Acquired hypothalamic obesity	Rapid weight gain after hypothalamic tumor, surgery, radiotherapy, trauma, or inflammation; reduced energy expenditure ± hyperphagia [22,38]	Setmelanotide under current indication; treat pituitary deficits; multidisciplinary neuroendocrine care [22,38]	Monitor adrenal and sodium disorders, particularly with central diabetes insipidus [38]

Abbreviations: LEP, leptin gene; LEPR, leptin receptor; POMC, pro-opiomelanocortin; PCSK1, proprotein convertase subtilisin/kexin type 1; MC4R, melanocortin-4 receptor; VUS, variant of uncertain significance.

### 6.3. From Rare Disease to Common Obesity

Rare disorders establish causal pathways that may be therapeutically relevant in common obesity, but the translation is not linear. Common obesity is highly polygenic, with thousands of small-effect variants interacting with environment and development. Polygenic risk scores can identify marked inherited susceptibility and divergent weight trajectories, but limited portability across ancestries, calibration, and incremental value over clinical history constrain present clinical use [61]. At present, polygenic scores should not be used to deny treatment or promise response.

Pharmacogenomics is similarly immature. Candidate associations with metformin transporters, central appetite genes, or drug-metabolizing enzymes have not yet produced robust prescribing rules for anti-obesity medicines in children [62,63]. Precision medicine must therefore distinguish actionable monogenic diagnoses from exploratory predictors in common obesity.

## 7. Building a Precision Phenotype for Common Childhood Obesity

### 7.1. A Multidimensional Baseline Assessment

A useful precision phenotype is constructed across several axes:

1. Developmental context: age, pubertal stage, height velocity, bone health, menstrual and reproductive considerations, neurodevelopment, and capacity for self-management;
2. Appetite biology: hunger intensity, loss-of-control eating, meal size, food preoccupation, nocturnal eating, and response to structured meals;
3. Energy expenditure and activity: resting energy expenditure when clinically indicated, sleep, mobility, cardiorespiratory fitness, and hypothalamic injury;
4. Metabolic risk: glucose tolerance, glycated hemoglobin (HbA1c), lipids, blood pressure, liver enzymes, liver fat/fibrosis assessment, sleep apnea, and polycystic ovary syndrome;
5. Body composition and fat distribution: waist-to-height ratio, visceral and hepatic fat where available, lean mass, strength, and sarcopenic risk;
6. Etiology and modifiers: onset and trajectory, family history, genetic/syndromic features, endocrine disease, and weight-promoting medication;
7. Treatment context: previous response, tolerability, route preference, injection burden, pregnancy risk, mental health, affordability, and continuity of supply. Screening should explicitly include depression, anxiety, loss-of-control or binge eating, restrictive eating, compensatory behaviors, weight stigma, and family stress, with developmentally appropriate tools and referral when indicated [10,64].

This multidimensional assessment is more informative than BMI plus a comorbidity count and helps explain why two children may reasonably receive different first-line medicines.

### 7.2. Biomarkers and Multi-Omics

Candidate biomarkers include fasting and postprandial gut hormones, leptin, adiponectin, insulin and C-peptide dynamics, lipid species, amino acids, inflammatory proteins, bile acids, microbiome features, metabolomics, and transcriptomic signatures. Imaging can quantify liver fat, visceral adiposity, and muscle composition. Wearable devices can characterize sleep, activity, and heart rate, while ecological momentary assessment can capture hunger and eating episodes.

Few candidate biomarkers have demonstrated clinical utility for selecting therapy. Many correlate with obesity severity but do not predict differential drug response. A useful predictor must be reproducible, improve decisions beyond readily available clinical information, and undergo prospective validation in diverse populations. Multi-omics models also risk overfitting, ancestry bias, and poor interpretability. Pediatric trials should embed biobanking, prespecified validation, and interoperable data standards [60,65].

### 7.3. Early Response as a Dynamic Biomarker

The most immediately actionable precision tool is a structured therapeutic trial. Baseline phenotyping generates a mechanism-based hypothesis—early response—then updates it. Hunger, meal size, adverse effects, adherence, dose achieved, BMI trajectory, waist, blood pressure, glucose, and liver markers should be reviewed at defined intervals. A child who reports a major reduction in food preoccupation but little early weight change may need more time or an evaluation of energy expenditure. A child with no appetite changes at adequate exposure may need a different pathway. A child with improved glycemia and liver enzymes despite modest BMI reduction may be deriving meaningful metabolic benefit.

Stopping rules should incorporate benefit, treatment burden, and the original therapeutic goal rather than rely on weight alone. Treatment should also not continue indefinitely without evidence of biological or patient-important benefit. Product-specific stopping rules remain controlling; for example, the liraglutide label requires reassessment of pediatric BMI response after 12 weeks on maintenance treatment, and phentermine/topiramate has staged BMI-response thresholds [33,34]. Multidomain benefit should complement, rather than override, labeled safety and stopping requirements.

### 7.4. Phenotype-to-Strategy Hypotheses

Adult proof-of-concept suggests that phenotype-guided selection of anti-obesity medication can improve average response, but the approach has not been prospectively validated in children and remains hypothesis-generating [66]. Table 4 presents candidate phenotype-to-strategy matches, and Figure 2 places these hypotheses within an iterative clinical framework. Figure 2 makes the feedback loop explicit: baseline assessment and mechanism characterization precede selection; exposure and response are measured before adaptation; and developmental change prompts reassessment. Mental health and eating behavior are baseline and longitudinal domains [10,64]. Mechanism matching in common obesity remains a hypothesis [66]. Treatment selection and response assessment must follow pediatric guidance [10,59], product-specific prescribing requirements [32,33,34], and the limitations of available response-prediction evidence [37].

**Table 4:** Hypothesis-generating phenotype-to-strategy matching framework.

Dominant Phenotype	Mechanism-Based Strategy	Measures that Should Drive Adaptation	Important Caveat
Marked hyperphagia/food preoccupation	Strong central appetite pathway treatment; test for monogenic or hypothalamic disease when indicated [11,19,59]	Hunger scores, food-seeking behavior, family burden, BMI trajectory [10,19,59]	Exclude eating disorder pathology and address environmental food insecurity [10,64]
Severe insulin resistance/dysglycemia	Incretin-based therapy; future GIP/GLP-1 approaches; optimize diabetes prevention [7,14,46]	OGTT/HbA1c, insulin sensitivity proxies, acanthosis, triglycerides [10,18]	Fasting insulin alone is an imprecise treatment target [18,59]
MASLD/visceral adiposity	Therapy with strong weight and liver-fat effects; future glucagon co-agonist or FGF21 strategy [7,23,24,25,51]	ALT trend, MRI-PDFF where available, fibrosis assessment, waist [67]	Normal ALT does not exclude MASLD; pediatric FGF21 data are not established [23,24,25,67]
Low satiation/large meals despite GLP-1	Future amylin combination or switch strategy [52,53,54,55]	Meal size, hunger/satiation, tolerability, nutritional intake [52,53,54,55]	Combination superiority and pediatric safety require trials [54,55]
Acquired hypothalamic obesity	MC4R pathway therapy plus pituitary replacement and energy expenditure support [22,38]	BMI, hunger, resting expenditure, adrenal/sodium safety, quality of life [22,38]	Endocrine deficits and neuro-oncology sequelae require parallel management [38]
Rare leptin–melanocortin disorder	Genotype/biomarker-directed replacement or receptor agonism (MC4R-directed treatment [19,20,21,38]; leptin replacement [26,27,28,29])	Hunger, growth, endocrine function, genotype-specific outcomes (MC4R-directed treatment [19,20,21,38]; leptin replacement [26,27,28,29])	Variant causality and pathway position determine treatment logic [38,59]
High lean-mass or nutritional vulnerability	Slower titration, protein/resistance exercise prescription, body composition monitoring [10]	Lean mass, strength, growth velocity, diet quality [10]	Percentage weight loss alone may reward an unsafe response [10]

Abbreviations: BMI, body mass index; GLP-1, glucagon-like peptide-1; GIP, glucose-dependent insulinotropic polypeptide; MC4R, melanocortin-4 receptor; FGF21, fibroblast growth factor 21; MASLD, metabolic dysfunction-associated steatotic liver disease; OGTT, oral glucose tolerance test; HbA1c, glycated hemoglobin; ALT, alanine aminotransferase; MRI-PDFF, magnetic resonance imaging proton-density fat fraction. Evidence note: references identify the mechanistic, trial, or guidance basis for each entry. Phenotype matching, advanced monitoring, and disease-modifying interpretations are proposed syntheses, not source-validated pediatric prescribing rules. Adult-only evidence is not a pediatric recommendation.

**Figure 2:**
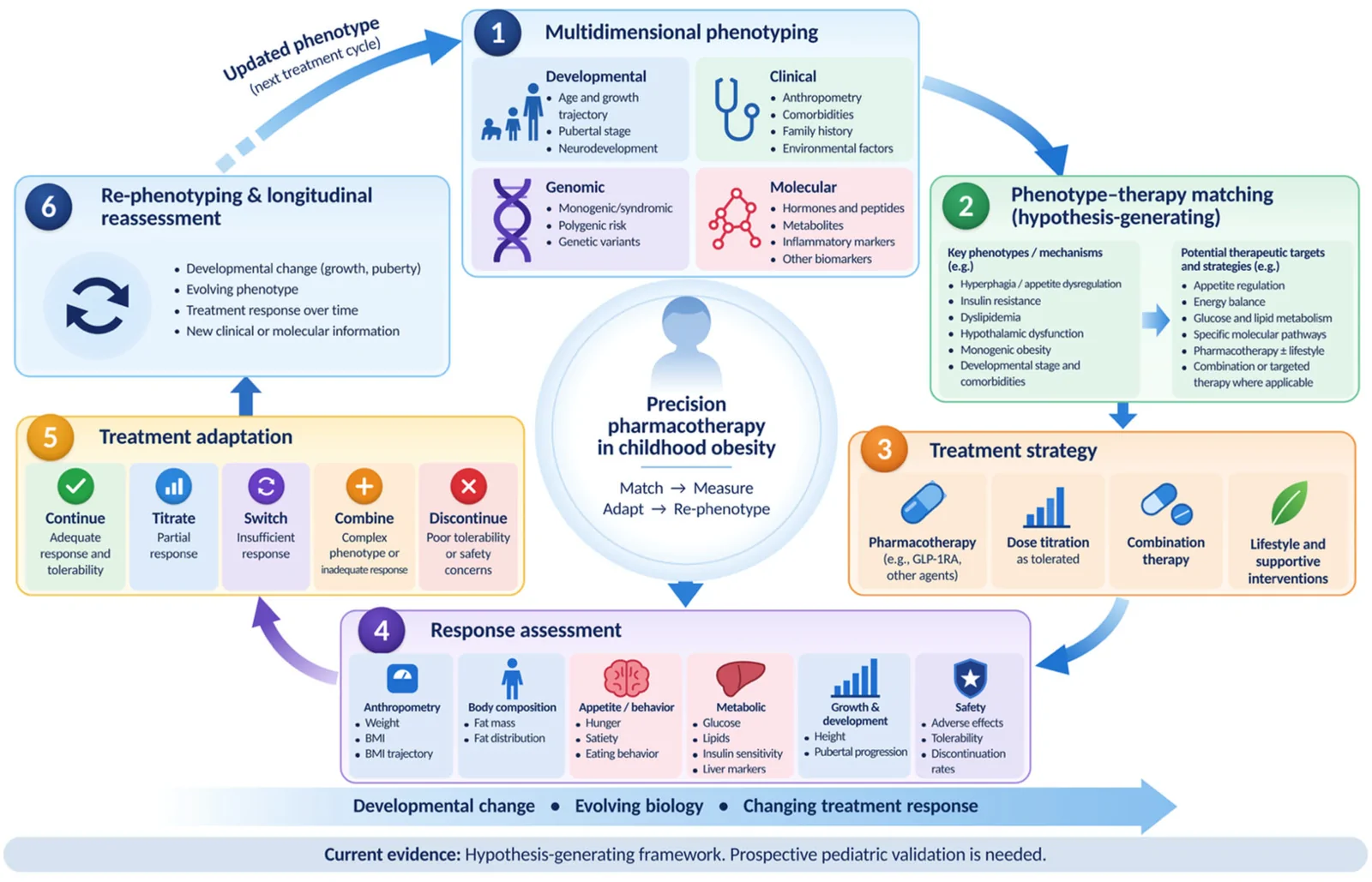
Dynamic framework for precision pharmacotherapy in childhood obesity. Multidimensional developmental, clinical, genomic, and molecular phenotyping informs hypothesis-generating phenotype–therapy matching and selection of an initial strategy. Response is evaluated across anthropometry, body composition, appetite and behavior, metabolic health, growth and development, and safety. Findings guide continuation, titration, switching, combination, or discontinuation, followed by longitudinal re-phenotyping as biology and treatment response change. Prospective pediatric validation of the framework remains necessary. Abbreviation: GLP-1RA, glucagon-like peptide-1 receptor agonist. This is an original synthesis, not formal expert consensus. Sources by component: pediatric assessment and longitudinal care [10,59]; indication-specific treatment and prescribing requirements [32,33,34,38]; response assessment [37]; eating-disorder considerations [64]; adult phenotype-guided proof-of-concept [66].

## 8. Redefining Disease Modification in a Growing Child

### 8.1. Weight Is an Intermediate Outcome

BMI reduction is clinically relevant and remains necessary for efficacy trials, but it does not fully define success. Disease modification should refer to altering the probability or timing of important downstream outcomes while preserving healthy development. The target depends on baseline risk. For a child with prediabetes and declining beta cell compensation, preventing type 2 diabetes may be central. For a child with MASLD, reducing liver fat and preventing fibrosis may matter most. For congenital leptin deficiency, restoration of pubertal and immune function forms part of causal correction. Figure 3 summarizes this life-course concept. Figure 3 illustrates proposed divergent life-course trajectories, rather than a proven reversal of a fixed disease course; its arrows are conceptual, not quantitative or causal estimates. Improvements in liver fat, glycemia, or appetite must be interpreted alongside growth, nutrition, and function; prevention of adult complications requires long-term follow-up rather than inference from short-term BMI reduction [7,10,29,67,68].

### 8.2. Growth, Puberty, and Bone and Lean Mass

Pediatric treatment should preserve height velocity and appropriate pubertal progression. Rapid appetite suppression can reduce energy, protein, iron, calcium, and vitamin D intake. Lean mass typically falls during weight loss even when fat mass accounts for most of the change. The clinical meaning depends on strength, maturation, and baseline body composition. Trials should report absolute and relative lean mass, appendicular lean mass where feasible, grip strength or functional measures, dietary protein, and resistance activity. Bone mineral accrual and fracture outcomes require longer follow-up than most current trials.

### 8.3. Hepatic and Visceral Adiposity

MASLD can progress from steatosis to steatohepatitis, fibrosis, and later cirrhosis, but pediatric natural history is heterogeneous. Liver enzymes are accessible but insensitive. MRI proton-density fat fraction provides a quantitative research endpoint, while elastography and validated fibrosis pathways can enrich higher-risk cohorts [5,67]. A treatment that reduces liver fat and fibrosis risk may be disease-modifying even when BMI reduction is less striking than with an appetite-dominant drug.

### 8.4. Beta Cell Preservation and Prevention of Type 2 Diabetes

Adolescence may offer a limited window in which reducing insulin demand and ectopic fat preserves beta cell reserve. Required trials are long and large, but intermediate designs can measure oral-glucose-tolerance-derived disposition indices, continuous glucose metrics in high-risk participants, and progression from normal glucose tolerance to prediabetes or diabetes. Adult cardiovascular-outcome evidence with semaglutide demonstrates that obesity treatment can reduce hard outcomes, but pediatric benefit cannot be assumed and will require life-course follow-up [68].

### 8.5. Psychosocial and Functional Health

Quality of life, stigma, depression, anxiety, school participation, sleep, mobility, and family burden are integral treatment outcomes. Severe hyperphagia can dominate family life, and reducing it may be clinically important before a large BMI change occurs. Conversely, pharmacotherapy may cause harm if it reinforces weight stigma or triggers restrictive eating. Structured pediatric obesity treatment with a dietary component has not been associated with increased eating disorder risk in aggregate, although individual screening remains essential [64]. Patient-reported outcomes should be developmentally validated, and trials should include adolescents with neurodevelopmental and socioeconomic diversity. Table 5 organizes the proposed multidomain outcomes.

**Table 5:** Proposed multidomain outcomes for disease modification in childhood obesity.

Domain	Core Outcome	Mechanistic or Advanced Outcome	Disease-Modifying Interpretation
Adiposity	Percentage BMI change; percentage of the 95th percentile; waist [7,10]	DXA/MRI fat mass, visceral fat [7,10]	Durable reduction in harmful adipose burden without impaired growth [10]
Development	Height velocity, Tanner stage, menstrual history [10,59]	Bone age, bone density/geometry, nutritional biomarkers [10,59]	Normal growth, puberty, and bone accrual preserved [10,59]
Lean tissue	Weight and BMI cannot resolve this domain [10]	DXA lean mass, muscle imaging, strength, physical function [10]	Fat loss favored while muscle function and protein adequacy are maintained [10]
Glycaemia	HbA1c, fasting glucose, diabetes incidence [6,18]	OGTT-derived beta cell function, continuous glucose, clamp subsets [18]	Delay or prevention of dysglycemia and preservation of beta cell compensation [6,18]
Liver	ALT/AST, clinical MASLD assessment [67]	MRI-PDFF, elastography, fibrosis biomarkers/histology in selected cases [67]	Reduced steatosis and prevention or regression of fibrosis [67]
Cardiovascular	Blood pressure and lipids [10,68]	Vascular function, cardiac structure, long-term events [68]	Lower cumulative vascular risk across the life course [68]
Appetite/behavior	Hunger, loss-of-control eating, food preoccupation [10,64]	Ecological assessment, eating microstructure [64,66]	Normalization of pathological drive without inducing disordered restriction [10,64]
Patient experience	Quality of life, treatment burden, school and family function [10]	Preference-based and caregiver outcomes [10]	Sustainable benefit that matters to the child and family [10]
Durability	Response during treatment [8,43,44,45]	Outcomes after interruption, switching and transition to adult care [43,44,45]	Persistent risk reduction, whether treatment is continued or safely de-escalated [43,44,45]

Abbreviations: BMI, body mass index; DXA, dual-energy X-ray absorptiometry; MRI, magnetic resonance imaging; OGTT, oral glucose tolerance test; HbA1c, glycated hemoglobin; ALT, alanine aminotransferase; AST, aspartate aminotransferase; MASLD, metabolic dysfunction-associated steatotic liver disease; MRI-PDFF, magnetic resonance imaging proton-density fat fraction. Evidence note: references identify the mechanistic, trial, or guidance basis for each entry. Phenotype matching, advanced monitoring, and disease-modifying interpretations are proposed syntheses, not source-validated pediatric prescribing rules. Adult-only evidence is not a pediatric recommendation.

**Figure 3:**
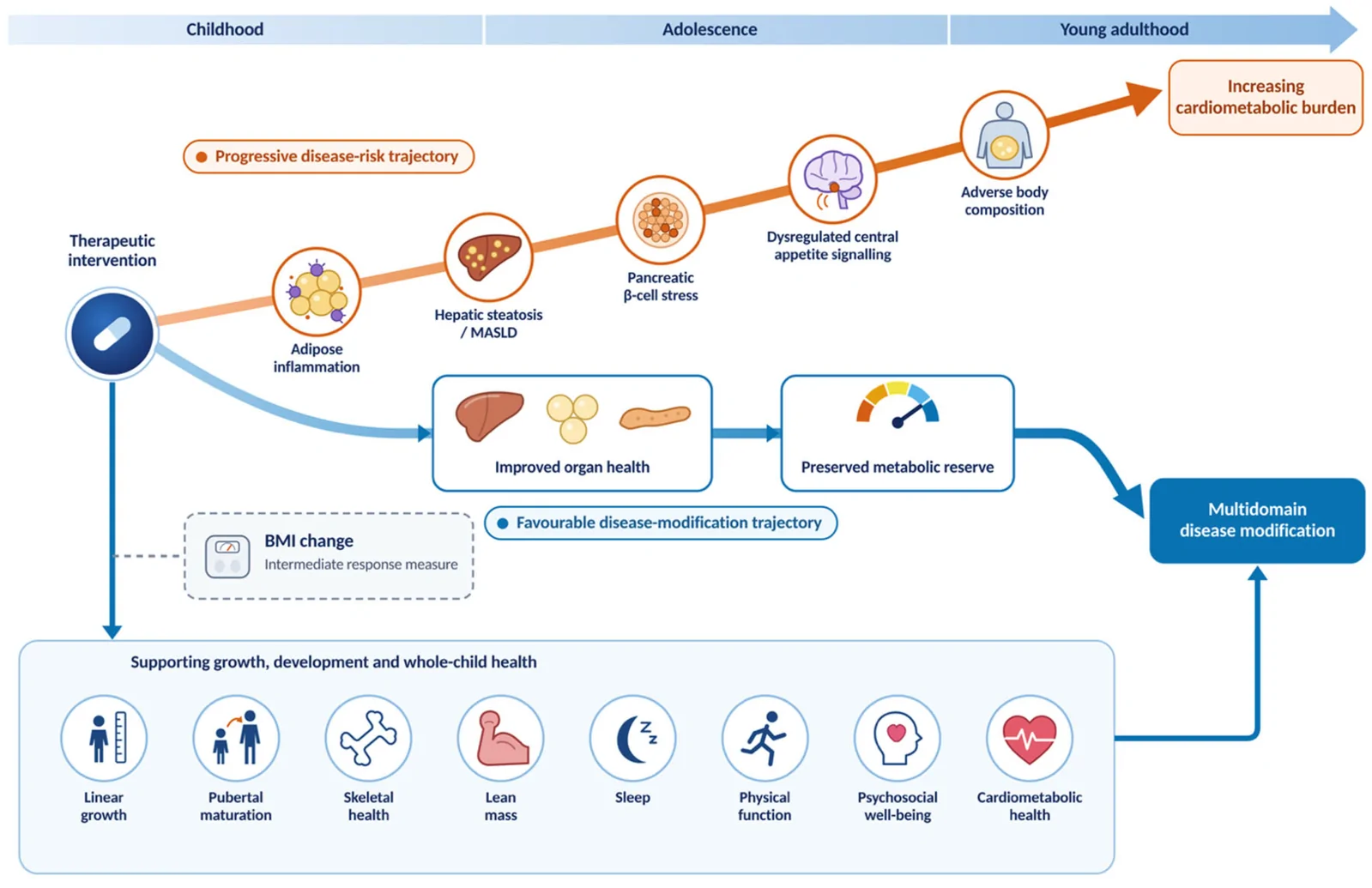
Mechanistic life-course framework for disease modification in childhood obesity. The upper trajectory links adipose inflammation, hepatic steatosis, beta cell stress, dysregulated central appetite signaling, and adverse body composition to increasing cardiometabolic burden. The lower trajectory depicts improved organ health and preserved metabolic reserve while supporting linear growth, pubertal maturation, skeletal health, lean mass, sleep, physical function, psychosocial well-being, and cardiometabolic health. BMI change is an intermediate response measure rather than a complete definition of disease modification. Abbreviation: MASLD, metabolic dysfunction-associated steatotic liver disease. These are proposed life-course trajectories rather than demonstrated pediatric prevention of future events [6,7,10,29,64,67,68].

## 9. A Dynamic Clinical Framework

The six-step cycle in Figure 2 is an original, hypothesis-generating synthesis developed for this review by integrating the 2023 American Academy of Pediatrics guideline [10], Endocrine Society guidance [59], product-specific prescribing requirements [32,33,34,38], response-prediction evidence [37], and adult phenotype-guided proof-of-concept [66]; it was not developed through a formal consensus process or prospectively validated in children. First, assess obesity severity, trajectory, complications, and functional burden, including urgent hyperglycemia, hypertension, and liver or sleep-related disease, to determine treatment intensity within comprehensive care [10,59,67]. Second, characterize developmental stage, appetite, metabolic risk, weight-promoting medications, and clues to monogenic or acquired hypothalamic disease [10,38,59]. At baseline, screen for eating disorders, depression, and anxiety, assess suicidality when indicated, and incorporate family context and appropriate psychological support; repeat these assessments during titration, at response reviews, and whenever symptoms arise [10,64]. Third, select an age- and indication-appropriate medicine using contraindications, expected benefit, family preferences, and access; use genotype- or lesion-directed treatment when the diagnosis meets its specific indication, and study emerging pediatric obesity agents within trials [10,32,33,34,38]. Fourth, review exposure, tolerability, adherence, nutrition, height velocity, pubertal progression, BMI trajectory, appetite, psychological well-being, and the relevant organ-specific goal at titration visits and subsequent response assessments. The proposed 3–6-month multidomain review is a pragmatic interval rather than a validated universal threshold; earlier product-specific stopping and escalation rules take precedence [32,33,34,37,38]. Fifth, continue, titrate, switch, or discontinue according to demonstrated benefit, labeled response requirements, safety, and treatment burden; combinations require supporting evidence and specialist oversight rather than extrapolation from adult efficacy [10,32,33,34]. Sixth, reassess phenotype and goals during puberty, after substantial weight change, with evolving complications or interrupted treatment, and during transition to adult care [10,59]. This cycle uses observed responses to update a therapeutic hypothesis while acknowledging that mechanism-guided selection for common childhood obesity remains unvalidated.

## 10. Safety, Ethics, and Equity

### 10.1. Developmental Pharmacovigilance

Long-term registries should link exposure to growth, pubertal development, pregnancy, gallbladder and pancreatic outcomes, renal events, mental health, eating disorders, bone outcomes, and body composition. Safety data should be stratified by age, sex, pubertal stage, neurodevelopmental status, ethnicity, and comorbidity. Because the newest agents may be used for many years, post-authorization evidence is as important as pre-authorization trials. A pragmatic class-specific monitoring framework is summarized in Table 6.

**Table 6:** Developmental safety and monitoring priorities for pediatric obesity pharmacotherapy.

Therapy or Class	Baseline Safeguards	Early Monitoring	Long-Term Monitoring
Orlistat	GI history; diet quality; fat-soluble vitamin risk [35]	GI tolerance; adherence; dietary restriction [35]	Fat-soluble vitamins; growth and nutritional adequacy [35]
Phentermine or phentermine/topiramate ER	Blood pressure; heart rate; mood and cognition; pregnancy potential; interacting stimulants [34,36]	Heart rate and blood pressure; insomnia; anxiety; cognition; pregnancy prevention [34,36]	Growth; mood; misuse risk; reproductive counseling; treatment persistence [34,36]
Liraglutide or semaglutide	GI and gallbladder history; hydration risk; diabetes medicines; pregnancy potential; nutrition [32,33]	GI tolerance; hydration; glucose when indicated; protein and micronutrient intake [32,33]	Growth and puberty; gallbladder and pancreatic events; body composition; eating disorder symptoms; pregnancy planning [7,8,32,33]
Emerging polyagonist or amylin strategy	Trial eligibility; glucose status; hepatic and cardiovascular phenotype; nutritional vulnerability (FGF21 analogues [23,24,25]; glucagon-containing polyagonists [49,50,51]; amylin combinations [52,53,54,55])	GI effects; glucose; heart rate; amino acid and protein adequacy; lean-mass trajectory (FGF21 analogues [23,24,25]; glucagon-containing polyagonists [49,50,51]; amylin combinations [52,53,54,55])	Growth, puberty, bone, and lean tissue; liver outcomes; class-specific post-authorization safety (FGF21 analogues [23,24,25]; glucagon-containing polyagonists [49,50,51]; amylin combinations [52,53,54,55])
Setmelanotide	Confirm indication; mood history; full skin examination; pituitary, adrenal, and sodium status in acquired hypothalamic obesity [38]	GI effects; pigmentation and nevi; mood and sexual adverse effects; adrenal or sodium complications when relevant [38]	Growth and puberty; skin surveillance; mental health; endocrine sequelae of the underlying disorder [38]
Leptin replacement	Confirm biochemical and molecular diagnosis; metabolic and hematological assessment; specialist access pathway [26,27,28,29]	Appetite and weight trajectory; metabolic response; injection reactions; anti-drug antibodies when indicated [26,27,28,29]	Growth, puberty, and immune function; metabolic durability; indication-specific lymphoma and antibody surveillance [26,27,28,29]

Abbreviations: GI, gastrointestinal; ER, extended release. Evidence note: references identify the mechanistic, trial, or guidance basis for each entry. Phenotype matching, advanced monitoring, and disease-modifying interpretations are proposed syntheses, not source-validated pediatric prescribing rules. Adult-only evidence is not a pediatric recommendation.

### 10.2. Reproductive Health and Mental Health

Adolescents who can become pregnant require confidential, developmentally appropriate counseling. Phentermine/topiramate is teratogenic and requires strict pregnancy prevention; rapid weight loss and delayed gastric emptying can affect reproductive planning and oral drug absorption. Mood, suicidality, anxiety, substance use, and eating disorder symptoms should be assessed without assuming that they are caused by or preclude pharmacotherapy. Setmelanotide requires specific monitoring for depression/suicidal ideation, sexual adverse effects, and pigmented lesions; acquired hypothalamic obesity also requires attention to adrenal and sodium disorders [38]. The FDA’s January 2026 review found no increased risk of suicidal ideation or behavior with GLP-1 receptor agonists and requested removal of that warning from relevant obesity-drug labels. Psychological screening remains appropriate in pediatric obesity care, but it should not imply a demonstrated causal GLP-1 class effect; this finding is distinct from setmelanotide- and topiramate-specific precautions [69].

### 10.3. Access and Algorithmic Fairness

Precision medicine can widen inequity if it becomes a premium service built on expensive imaging, sequencing, and continuous sensors. Clinically useful precision care begins with information available in ordinary clinics: trajectory, family history, hyperphagia, pubertal stage, complications, treatment preference, and early response. Genetic testing and targeted drugs should be accessible when indicated. Predictive algorithms require training and validation across ancestries and socioeconomic groups to avoid reproducing historical underdiagnosis and undertreatment.

Weight stigma also affects evidence. Families may delay care after repeated blame, and clinicians may under-treat obesity while aggressively treating its consequences. Shared decision-making should describe obesity biology, expected benefit, uncertainty, burden, and chronicity in neutral language.

## 11. Mechanistic and Clinical Validation of Precision Pharmacotherapy

### 11.1. Mechanism-Enriched Pediatric Trials

Future trials should deliberately enroll children with predefined phenotypes such as severe hyperphagia, MASLD, acquired hypothalamic obesity, insulin-resistant dysglycemia, or low satiation. This does not require excluding broad populations; it requires prespecified interaction analyses and sufficient sample sizes to identify differential response.

### 11.2. Comparative and Adaptive Designs

Placebo-controlled efficacy is no longer the only relevant question. Head-to-head trials should compare established high-efficacy medicines, while sequential multiple-assignment designs can test when to switch or add a second mechanism. Early-response algorithms should be prospectively evaluated rather than derived after the fact.

### 11.3. Development and Validation of a Pediatric Core Outcome Set

A minimum outcome set should include percentage BMI change, height velocity, pubertal status, fat and lean mass, blood pressure, glycemia, lipids, hepatic assessment, hunger, quality of life, nutritional adequacy, adverse effects, and treatment persistence. High-risk trials should add beta cell function, MRI liver fat, fibrosis assessment, sleep, and physical function.

### 11.4. Biobanks and Interoperable Data

Trials and registries should collect DNA, plasma/serum, stool where relevant, imaging, and longitudinal digital phenotypes under harmonized protocols. Replication cohorts must be planned at inception. Data governance should protect children while enabling clinically useful discovery.

### 11.5. Life-Course Durability

Pediatric studies should include planned follow-up through transition to adult care. Key questions are whether early treatment prevents diabetes and MASLD, whether lean mass and bone accrue normally, how treatment can be safely paused for pregnancy or other reasons, and whether an early period of pathway correction changes later treatment intensity. These outcomes cannot be inferred from one-year weight curves.

### 11.6. Limitations of This Review

This narrative review is intended to integrate molecular rationale and clinical translation, not to estimate comparative treatment effects. Study selection was not conducted with duplicate screening or formal risk-of-bias assessment, and evidence for several emerging classes is derived predominantly from adult trials. Regulatory status and late-stage pipelines are changing rapidly. The phenotype-to-strategy framework should therefore be regarded as a testable clinical model rather than a validated prescribing algorithm.

## 12. Conclusions

Pediatric obesity pharmacotherapy acts on defined endocrine and neural pathways, but clinical readiness varies substantially. Established options for nonsyndromic obesity include age-appropriate orlistat, short-term phentermine, phentermine/topiramate, liraglutide, and injectable semaglutide; setmelanotide has diagnosis- and age-specific indications for selected genetic disorders and acquired hypothalamic obesity [10,32,33,34,35,36,38]. Specialist leptin replacement can produce substantial benefits in congenital leptin deficiency, although access and regulatory context differ from generalized lipodystrophy [26,27,28,29]. These rare-disease observations illustrate targeted pathway treatment, but small cohorts and limited long-term follow-up restrict conclusions about lifelong disease modification. GIP/GLP-1 agonists [46,47,48], glucagon-containing polyagonists [49,50,51], amylin combinations [52,53,54,55], and FGF21 analogues [23,24,25] provide complementary molecular hypotheses supported mainly by adult studies; these data do not establish routine pediatric obesity indications or developmental safety.

Precision care should integrate the child’s developmental, metabolic, appetite, and psychosocial phenotype with labeled indications, treatment preference, and early response. Long-term safety evidence remains incomplete, particularly for growth, puberty, bone accrual, and lean tissue. Gastrointestinal adverse effects are established concerns with incretin therapy [7,8,32,33]; psychiatric risks and monitoring requirements differ by medicine and should not be assumed to represent a uniform causal class effect [32,33,34,38]. Eating disorders, depression, and anxiety require attention before and during treatment [10,64]. Weight recurrence after interruption or discontinuation also limits durability and requires planning for ongoing care [43,44,45]. The proposed framework and multidomain outcomes are testable clinical models rather than validated prescribing rules or proof of a pediatric cure. Progress requires comparative pediatric trials and longitudinal follow-up demonstrating sustained benefit while preserving nutrition, development, function, and psychological well-being.

## Data Availability

No new data were created or analyzed in this study.
